# Experimental evolution of *Vibrio cholerae* identifies hypervesiculation as a way to increase motility in the presence of polymyxin B

**DOI:** 10.3389/fmicb.2022.932165

**Published:** 2022-08-22

**Authors:** Sean Giacomucci, Annabelle Mathieu-Denoncourt, Antony T. Vincent, Hanen Jannadi, Marylise Duperthuy

**Affiliations:** ^1^Département de Microbiologie, Infectiologie et Immunologie, Université de Montréal, Montréal, QC, Canada; ^2^Département des Sciences Animales, Faculté des Sciences de l’Agriculture et de l’Alimentation, Université Laval, Québec, QC, Canada; ^3^Institut de Biologie Intégrative et des Systèmes, Université Laval, Québec, QC, Canada

**Keywords:** *Vibrio cholera*, flagella, motility, membrane vesicles, experimental evolution, polymyxin, subinhibitory concentration, resistance

## Abstract

*Vibrio cholerae* includes strains responsible for the cholera disease and is a natural inhabitant of aquatic environments. *V. cholerae* possesses a unique polar flagellum essential for motility, adhesion, and biofilm formation. In a previous study, we showed that motility and biofilm formation are altered in the presence of subinhibitory concentrations of polymyxin B in *V. cholerae* O1 and O139. In this study, we performed an experimental evolution to identify the genes restoring the motility in the presence of a subinhibitory concentration of polymyxin B. Mutations in five genes have been identified in three variants derived from two different parental strains A1552 and MO10: *ihfA* that encodes a subunit of the integration host factor (IHF), *vacJ* (*mlaA*) and *mlaF*, two genes belonging to the maintenance of the lipid asymmetry (Mla) pathway, *dacB* that encodes a penicillin-binding protein (PBP4) and involved in cell wall synthesis, and *ccmH* that encodes a c-type cytochrome maturation protein. We further demonstrated that the variants derived from MO10 containing mutations in *vacJ*, *mlaF*, and *dacB* secrete more and larger membrane vesicles that titer the polymyxin B, which increases the bacterial survival and is expected to limit its impact on the bacterial envelope and participate in the flagellum’s retention and motility.

## Introduction

*Vibrio cholerae* is a gram-negative bacterium, and the strains belonging to the O1 and O139 serogroups are responsible for the cholera disease, which infects millions of people per year worldwide. More than 200 serotypes have been reported but only the O1 and O139 cause cholera (Harris et al., 2012). The O1 strains are further divided into 2 biotypes: classical and El Tor. The classical biotype was responsible for six pandemics and has been replaced by the El Tor biotype in 1961, which is responsible for the ongoing 7th pandemic (Harris et al., 2012). The O139 serotype, which causes local epidemics, appeared in 1992 and is derived from the O1 El Tor biotype (Faruque et al., 2003). Some strains from non-O1/non-O139 serogroups can cause less serious gastroenteritis (Aydanian et al., 2011).

For most pathogenic bacteria, motility is essential for host colonization and virulence (Josenhans and Suerbaum, 2002). Different types of motility exist: swimming, swarming, twitching, gliding, and sliding, the latest being a passive process that does not involve energy (Kearns, 2010). Swarming, twitching, gliding, and sliding are the surface motility mechanisms whereas swimming allows bacteria to move in liquid suspensions and involves active propulsion by the flagella. Flagella are filamentous apparatus anchored to the bacterial envelope and are divided into three major structures: the basal body, the hook, and the filament (Echazarreta and Klose, 2019). The movement is ensured by the rotation of the flagellum’s filament driven by a motor-like structure. In most bacteria, the motor uses an H + gradient as an energy source (Nakamura and Minamino, 2019). However, in *Vibrio*, the rotation of the flagellum is driven by a Na + motor (Kojima et al., 1999), which provides a rotation speed up to five times faster than the H + driven flagella of *E. coli* (Magariyama et al., 1994). *V. cholerae* possesses a single polar flagellum. Its filament is composed of five homologous subunits: FlaA, FlaB, FlaC, FlaD, and FlaE, and the flagellum is coated by an outer membrane sheath (Fuerst and Perry, 1988; Klose and Mekalanos, 1998). Besides its role in motility, *Vibrio’*s flagellum is also essential for biofilm formation, colonization, and virulence (Echazarreta and Klose, 2019). In *V. cholerae*, the expression of the flagellar genes is tightly regulated and is organized in a four-step hierarchical process, resulting in four classes of genes. The FlaA flagellin gene (*flaA*) belongs to the class II genes and is the only essential flagellin for motility (Klose and Mekalanos, 1998). The other flagellins (*flaB*, *flaC*, *flaD*, and *flaE*), belong to the class IV genes (Echazarreta and Klose, 2019).

Antimicrobial peptides (AMPs) are molecules of low molecular weight with antimicrobial activities against viruses, fungi, or bacteria (Boparai and Sharma, 2020). They can also modulate the immune response, as a part of innate immunity (Boparai and Sharma, 2020). AMPs are produced by both eukaryotic host cells and bacteria (Bevins and Salzman, 2011; Boparai and Sharma, 2020). Polymyxin B (PmB), an AMP produced by the gram-positive bacteria *Bacillus polymyxa*, has been revived as a last-resort treatment for multidrug-resistant gram-negative bacteria (Yang et al., 2020). Several mechanisms have been reported to explain the antimicrobial activity of AMPs, the best known being the electrostatic interaction with the bacterial envelope, leading to pore formation and cell death (Daugelavicius et al., 2000; Boparai and Sharma, 2020). There are a growing number of evidence showing that AMPs at subinhibitory concentrations can modulate the resistance and the virulence of gram-negative bacteria (Duperthuy, 2020), including our studies on *V. cholerae* (Duperthuy et al., 2013; Giacomucci et al., 2019; Mathieu-Denoncourt and Duperthuy, 2021). Because of their co-evolution with their host and with microbial competitors, the *Vibrio* have evolved mechanisms to resist AMPs that relate to lipopolysaccharides (LPS) modifications, outer membrane remodeling, induction of the envelope stress response, efflux of AMPs, repression of AMPs’ expression by the host cells, and titration by membrane vesicles (MVs) (Destoumieux-Garzón et al., 2014).

Bacteria produce MVs to remodel their membrane and release diverse molecules in the extracellular environment (Bohuszewicz et al., 2016; Toyofuku et al., 2019). In gram-negative bacteria, MVs are produced from blebbing of the bacterial envelope or by explosive cell lysis. Depending on their origin, MVs can contain LPS, peptidoglycan, proteins, lipids, and nucleic acids from extracellular, periplasmic, and cytoplasmic origins (Altindis et al., 2014; Toyofuku et al., 2019). MVs have a role in DNA exchange, virulence, and resistance to phages, plasmas, and antimicrobials (Guerrero-Mandujano et al., 2017). *V. cholerae* produces MVs through a VacJ/Yrb-dependent process (Roier et al., 2016). This system is implicated in the membrane phospholipids asymmetry and its inactivation increases MVs production (Roier et al., 2016). The MVs of *V. cholerae* can carry proteins implicated in virulence (Rompikuntal et al., 2015; Zingl Franz et al., 2021), can help bacterial surface modulation during the infection (Zingl et al., 2020), and have a role in AMPs resistance and cross-resistance (Duperthuy et al., 2013; Rompikuntal et al., 2015; Mathieu-Denoncourt et al., 2021).

In our previous study, we showed that the biofilm formation was impaired in the presence of subinhibitory concentrations of PmB, due to a significant reduction in the proportion of flagellated *V. cholerae* O1 and O139 (Giacomucci et al., 2019). Accordingly, a motility reduction was observed in the presence of PmB and was sometimes associated with a non-homogenous flower-like motility pattern on soft agar. In this study, we investigated the mechanisms explaining the motility restoration of some bacterial cells leading to the flower-like motility pattern in the presence of PmB. To do so, we performed an experimental evolution in the presence of PmB in two strains of *V. cholerae, i.e.*, O1 El Tor strain A1552 and O139 strain MO10. After sequencing, we identified mutations in genes involved in global regulation (*ihfA*), the outer membrane lipid asymmetry maintenance (*vacJ* and *mlaF*), and cell wall synthesis (*dacB*). We further demonstrated that the increased motility of the MO10 variants containing mutations in *vacJ*, *mlaF* and *dacB* is due to an increased titration of PmB through hypervesiculation and large vesicles production. The mechanism explaining the gain of motility resulting from the mutation in *ihfA* remains to be explored and is not linked to flagellins’ expression.

## Materials and methods

### Bacterial strains and growth conditions

Two *V. cholerae* strains have been used in this study: A1552 (O1, El Tor, Inaba) and MO10 (O139), initially isolated from patients in Peru and India, respectively (O’shea et al., 2004; Allué-Guardia et al., 2018). Bacterial growth was performed in Luria-Bertani (LB) medium supplemented with 25 μg/ml of PmB (PmB sulfate, Millipore-Sigma) (Giacomucci et al., 2019). Growth curves were performed as follows: precultures were started from overnight cultures (1:50) in 10 ml of fresh LB medium at 37°C to an OD_600 nm_ ∼0.2–0.4. They were then diluted to a final OD_600nm_ of 0.001 in LB supplemented with 0.2% L-arabinose when indicated. Growth was followed at 37°C by measuring the OD_600 nm_ each 5 min for 24 h using SpectramaxID3 Molecular Device. Between two readings, plates were constantly and vigorously shaken. All results correspond to, at least, two technical replicates within three biological replicates for each strain and condition.

### Complementation with pBAD24 vector

All expression plasmids for complementation were constructed from the multicopy pBAD24 vector (Guzman et al., 1995) and are listed in Table 1. Empty vector was extracted from *E. coli* DH5α using QIAprep Spin Miniprep Kit (#27106) from Qiagen. The complete open reading frames from the target genes were amplified by polymerase chain reaction (PCR) (Q5^®^ High-Fidelity DNA Polymerase, #M0491S, New England Biolab) with the respective pair of primers containing restriction sites listed in Table 2 and purified with Monarch^®^ PCR & DNA Cleanup Kit (#T1030L, New England Biolab). Vectors and amplicons were double-digested with *Hin*dIII-HF and *Eco*RI-HF (#R3104S and #R3101S, New England Biolab) or with *Hin*dIII-HF and *Xba*I (#R0145S New England Biolab) according to the manufacturer’s guidelines. Digested products were purified from electrophoresis gel (Monarch^®^ DNA Gel Extraction Kit, #T1020L, New England Biolab) and were then ligated with T4 DNA ligase (#M0202S, New England Biolab). Newly ligated plasmids were purified on columns (Monarch^®^ PCR & DNA Cleanup Kit, #T1030L, New England Biolab), then transformed by heat shock, and amplified in *E. coli* DH5α (Bryant, 1988). The inserts’ sequences were verified using the pBAD24-verif_Fwd and pBAD24-verif_rev primers. Purified plasmids from *E. coli* were then transformed by electroporation in *V. cholerae* MO10-WT or A1552-WT strains with 1.25 kV for 5 ms (O’callaghan and Charbit, 1990). Then, respective plasmids were transformed in *V. cholerae* A1552-V6 or MO10-V2 or –V8 (O’callaghan and Charbit, 1990).

**TABLE 1:** Bacterial strains and plasmids used in this study.

Strain/plasmid	General characteristics	References
*V. cholerae*		
A1552-WT	Wild-type strain, O1 El Tor Inaba, highly pathogenic strain isolated from human cholera infection.	Allué-Guardia et al., 2018
A1552-V6	Hypermotile variant derived from A1552. Deletion of 12 nucleotides in *ihfA* that results in the loss of 4 amino acids (67–70) and mutation of one amino acid in position 66 (K66N).	This study
MO10-WT	Wild-type strain, O139, isolated from human infection	O’shea et al., 2004
MO10-V2	Hypermotile variant derived from MO10. Point mutation resulting in the insertion of a stop codon in *mlaF* that leads to a loss of function. Deletion of 11 nucleotides in *dacB* resulting in a shift in the open reading frame, the emergence of a stop codon and the loss of ∼30% of DacB C-terminal residues.	This study
MO10-V8	Hypermotile variant derived from MO10. Deletion of 371 nucleotides in a region covering *ccmH* and *vacJ* (*mlaA*) genes.	This study
MO10Δ*vacJ:cmR*	*vacJ* knockout mutant through cmR (chloramphenicol resistance cassette) substitution	This study
*E. coli*		
DH5α	F– φ80*lacZ*ΔM15 Δ(*lacZYA-argF*)U169 *recA*1 *endA*1 *hsdR*17(r_K_^–^, m_K_^+^) *phoA supE*44 λ–*thi*-1 *gyrA*96 *relA*1	Tu et al., 2005
*Plasmids*		
pBAD24	Expression vector. Arabinose inducible promoter, resistance cassette to carbenicillin (https://www.addgene.org/vector-database/1845/)	Guzman et al., 1995
pBAD24-*ihfA*	pBAD24 vector with complete *ihfA* open reading frame from A1552	This study
pBAD24-*dacB*	pBAD24 vector with complete *dacB* open reading frame from A1552	This study
pBAD24-*mlaF*	pBAD24 vector with complete *mlaF* open reading frame from A1552	This study
pBAD24-*vacJ*	pBAD24 vector with complete *vacJ* open reading frame from A1552	This study
pDL1093	Temperature-sensitive mTn*10* delivery vector	Shull and Camilli, 2018
pCas9-cr4	Cas9 nuclease under control of pTet promoter	Reisch and Prather, 2017
pKDsgRNA-p15	Arabinose inducible lambda red and anhydrotetracycline inducible sgRNA expression that targets pCas9 plasmid	Reisch and Prather, 2017
pKDsgRNA-p15-kanR	Lambda red with *ara* promotor, a kanamycin resistance cassette	This study

**TABLE 2:** Primers used in this study.

Primer	Sequence (5′ to 3′)	Used for
pBAD24-verif_Fwd	GCGTCTTTTACTGGCTCTTCTC	Verification of constructions
pBAD24-verif_rev	TTGATGCCTGGCAGTTTATGG	Verification of constructions
*Eco*RI-*ihfA*-Fwd	AGTACCGAATTCTTATGGCGCTCACAAAGGC	Construction of pBAD24-*ihfA*
*ihfA*-*Hin*dIII-rev	ATCATTAAGCTTGCTCGGTCTTATTTTTCGACT	Construction of pBAD24-*ihfA*
*vacJ*-*Xba*I-rev	ATCATTTCTAGAATTTCAGAGACCATCATAAC	Construction of pBAD24-*vacJ*
*Eco*RI-*vacJ*-Fwd	AGTACCGAATTCATGGTTGGCCGCTTATCT	Construction of pBAD24-*vacJ*
*Eco*RI-*dacB*-Fwd	AGTACCGAATTCATGCTTTTTCGCTTCATACCT	Construction of pBAD24-*dacB*
*dacB*-*Hin*dIII-rev	ATCATTAAGCTTCCTAGTTTGGCTTAGATGCC	Construction of pBAD24-*dacB*
*cmr*-fwd	ACGTTGATCGGCACGTAAGAGGTTCCAACTTTCACC	Construction of MO10Δ*vacJ:cmR*
*cmr*-rev	TGGATTCTCACCAATAAAAAACGCCCGGCGGC	Construction of MO10Δ*vacJ:cmR*
*vacJ*-up-Fwd	TCAGCATGTTCAGGCTTGGC	Construction of MO10Δ*vacJ:cmR*
*vacJ*-up-rev	CGTGCCGATCAACGTACGTAATTCCATGTGCAAAGGC	Construction of MO10Δ*vacJ:cmR*
*vacJ*-dwn-Fwd	ATTGGTGAGAATCCATGGTCTCTGAAATCAGGCTGAC	Construction of MO10Δ*vacJ:cmR*
*vacJ*-dwn-rev	AGCCATTCGCTCTATGTCCAA	Construction of MO10Δ*vacJ:cmR*
kanr R_xhoI	AGCTCTCGAGCTAAAACAATTCATCCAGTA	Construction of pKDsgRNA-p15-kanR
kanr F_xbaI	AGCTTCTAGAGTTTGATTTTTAATGGATAATGTG	Construction of pKDsgRNA-p15-kanR
pKDsg verif F	TTGCCGTCACTGCGTCTTT	Construction of pKDsgRNA-p15-kanR
pKDsg verif R	CCAGCTCTGAGCCTCAAGA	Construction of pKDsgRNA-p15-kanR

### Motility assays

Motility assays were performed as previously described (Giacomucci et al., 2019). Briefly, bacteria were spotted on LB plates containing 0.3% agar (soft agar) and incubated for 24–48 h at 37°C. The motility was evaluated by measuring the diameter of bacterial growth. PmB was added to the plate at a concentration of 25 μg/ml when indicated. The selection of variants was performed on technical triplicates. Experiments using variants were performed in biological triplicates. Statistical analyses were performed using a one-way analysis of variance (ANOVA) test (*p* < 0.05).

### Sequencing, genome assembly, and analysis

The DNA extraction of the bacterial strains was performed using Promega Wizard Genomic DNA Purification Kit according to the manufacturer’s guidelines. The wild-type strains MO10 and A1552 were sequenced by PacBio RS II and Illumina MiSeq at the Génome Québec Innovation Center (Centre Hospitalier Universitaire Sainte-Justine, Montréal, QC, Canada). The PacBio sequencing reads were assembled with Flye 2.8.1 (Kolmogorov et al., 2019). The sequences corresponding to chromosomes I and II were circularized with various tools from the EMBOSS version 6.6.0 suite (Rice et al., 2000). Illumina reads were subsequently used to polish assemblies with BWA version 0.7.17-r1188 (Li, 2013), SAMtools version 1.12 (Li et al., 2009), and Pilon version 1.23 (Walker et al., 2014).

The DNA of the variants sequenced by Illumina MiSeq at the Génome Québec Innovation Center (McGill University, Montréal, QC, Canada). The reads were filtered using FASTP version 0.20.1 (Chen et al., 2018). The mutations were identified and validated in relation to the genomes of the parental strains, beforehand annotated with Prokka version 1.14.5 (Seemann, 2014), with both Snippy version 4.6.0 Seemann (2015) and Breseq version 0.35.2 (Barrick and Lenski, 2009; Barrick et al., 2009).

### Sequences homologies, alignments, and features analysis

*Escherichia coli* K12 MG1655 and *Vibrio cholerae* IhfA protein sequences’ identity and similarity were calculated with protein–protein BLAST (Altschul et al., 1997, 2005). Alignment of two or multiple sequences was respectively performed using semi-global alignment “glocal” (Brudno et al., 2003) and MUSCLE (Edgar, 2004) from Snapgene^®^. Figure 4B was edited from Easyfig graphical sequence alignment (Sullivan et al., 2011), and panels Figures 4A,C,D were edited from Snapgene^®^ alignments. The signal peptide was identified with protcompB (V9.0) (Protcompb) and SignalP 5.0 (Nielsen et al., 2019). Putative –35 and –10 promoter boxes were identified with BPROM service from Softberry (Solovyev and Salamov, 2011; Supplementary Figures S1–S3). *Vibrio cholerae* genes and protein sequence homologies were compared to *Escherichia coli* K12 MG1655 (NC_000913.3).

### Membrane vesicles isolation, protein content analysis, and lipopolysaccharides quantification

Membrane vesicles were isolated as previously described (Duperthuy et al., 2013). Briefly, *V. cholerae* wild-type strain MO10 and its hypermotile variants MO10-V2 and MO10-V8 were grown in 125 ml LB broth in 250-ml glass Erlenmeyer flasks for 16 h at 37°C with agitation. Bacterial cells were removed completely by centrifugation and filtration of the supernatant using acetate cellulose 0.2-μm filters (Millipore). MVs were recovered from 25 ml cell-free supernatant by ultracentrifugation at 100,000 × *g* for 3 h at 4°C in a 70-Ti rotor (Beckman Instruments) and suspended in 250 μl of phosphate-buffered saline (PBS).

The vesicle lipopolysaccharides (LPSs) were then quantified using Pierce Chromogenic Endotoxin Quant Kit according to the manufacturer’s instructions, and the protein content of the vesicles was quantified using a Bradford assay (Bio-Rad 500-0006) and a bovine serum albumin (BSA) standard curve. Data were obtained from at least 3 independent experiments done in technical duplicates. Statistical analysis was done using Student’s *t*-tests (*p* < 0.05).

### Survival in the presence of a lethal concentration of PmB

Midlog cultures of MO10-WT, MO10-V2, or MO10-V8 were incubated for 30 min at 37°C with 200 μg/ml of PmB. Afterward, 10 μl of 10-fold dilutions of the mixture was spotted on LB Agar (LBA), incubated for 18 h at 37°C, and numerated. For the MVs protection assays, 90 μl of a midlog culture of MO10 was incubated for 1 h at 37°C with 200 μg/ml of PmB, and 5 μl of the MVs preparations isolated from MO10-WT, MO10-V2, or MO10-V8. A final concentration representing 5 times the concentration of vesicles produced by an overnight culture has been used, and the MV concentrations have been normalized to the MO10-WT MV concentration ([norm]) according to the LPS dosage, or not. Similarly, 10-fold dilutions were spotted on LBA and numbered. Data are presented as relative survival percentage (CFU/ml of each condition with PmB divided by CFU/ml of the condition without PmB) of at least three independent experiments done in technical duplicates. Statistical analysis was performed using a one-way ANOVA test.

### Electron microscopy

The bacteria were visualized by transmitted electron microscopy (TEM Hitachi H-7100) as previously described (Giacomucci et al., 2019). Bacterial cultures were obtained by dilution 1:50 of overnight culture in a fresh LB medium, followed by incubation at 37°C until the culture reached an OD_600nm_ of 0.08. Bacteria in the exponential growth phase were then diluted in a fresh LB medium containing 25 μg/ml of PmB to an OD_600nm_ of ∼ 0.5. A volume of 50 μl of fresh bacterial culture was spread on formvar and carbon coated 200 Mesh hexagonal slim bar grids. Mounting was washed with 50 mM cacodylate acid saccharose 3% and labeled using 1% phosphotungstic acid for 2 s. Bacteria were visualized at 75 kV and pictures were obtained using a digital camera AMT XR-111 (Advanced Microscopy Techniques).

### Sequestration of the PmB by the membrane vesicles

To assess the sequestration of PmB by the MVs, 30 ml of the filtered supernatants from overnight cultures of MO10, MO10-V2, or MO10-V8 was incubated at 37°C for 1 h with or without 10 or 25 μg/ml of PmB (Sigma-Aldrich). Samples (1.5 ml) were taken directly after the incubation with PmB (T). Vesicles (V) were then isolated by ultracentrifugation. Samples (1.5 ml) from the vesicle-free supernatants (S) were taken, and then, the vesicles were suspended in 200 μl of PBS.

Proteins from total (T) and vesicle-free supernatants (S) were precipitated with 150 μl trichloro-acetic acid (100% w/v) for 16 h at 4°C. Samples were then centrifuged at 20,000 × *g* for 20 min at 4°C, and proteins were suspended in 20 μl of Laemmli buffer. For the vesicle fraction, 10 μl of the MVs preparation was suspended in 20 μl of Laemmli buffer. As a control, 5 μl of PmB at 20 mg/ml (Sigma-Aldrich) was suspended in 120 μl of Laemmli buffer. All the samples were boiled for 10 min at 100°C and 7 μl of each sample migrated on 13% sodium dodecyl sulfate (SDS) gels. Precision plus dual color protein ladder from Bio-Rad was used as a molecular weight scale. Gels were stained with Coomassie blue and silver nitrate 0.2%.

### Quantitative PCR analysis of the flagellins genes’ expression

RNA extraction, cDNA construction, and qPCR analysis were performed as described before (Giacomucci et al., 2019; Mathieu-Denoncourt and Duperthuy, 2021). *V. cholerae* strains A1552, MO10, and their variants were grown in LB to an OD_600nm_ of 0.4 at 37°C with agitation. The bacterial cells from 10 ml of culture were recovered by centrifugation at 5,500 × *g* and suspended in 1 ml TRIzol reagent (Invitrogen). Total RNAs were extracted according to the manufacturer’s instructions, and 500 ng of RNA was retrotranscribed to cDNA using QuantiNova Reverse Transcription Kit (Qiagen). RNA and cDNA were quantified with nanodrop and visualized on 2% agarose gel.

Flagellins’ expression (*flaA* and *flaE*) was assessed by qPCR using primers as previously described (Giacomucci et al., 2019) and normalized with the expression of *recA* as the housekeeping gene. The relative expression of *flaA* and *flaE* of A1552-V6 and MO10-V2 and –V8 was calculated compared to their expression in A1552-WT and MO10-WT, respectively. Flagellins genes were amplified using PerfeCTa SYBR Green FastMix Low ROX in QantStudio 3 real-time PCR system (Thermo Fisher) with a protocol consisting of 1 × 30 s at 95°C; 40 × 95°C for 5 s, 58°C for 17 s, and 70°C for 12 s.

### MO10Δ*vacJ*::cmR construction

A MO10 mutant lacking *vacJ* has been constructed through CmR substitution using the lambda red recombination system, as previously described (Yamamoto et al., 2009). Briefly, up and downstream regions of the target gene of approximatively 1,000 pb were amplified by PCR using primers listed in Table 2. The chloramphenicol resistance cassette (cmR) from pCas9 CR4 (Reisch and Prather, 2017), with its promotor and terminator, was also amplified by PCR. The cmR and the flanking regions of the target genes were bound together by overlap PCR and purified using Monarch DNA Gel Extraction Kit (NEB). A kanamycin resistance cassette (kanR) from pDL1093 (Shull and Camilli, 2018) was amplified using primers listed in Table 2, adding *Xho*I et *Xba*I restriction sites in 3′ and 5′. The purified amplicon and pKDsgRNA-p15 (Reisch and Prather, 2017) were digested using *Xho*I and *Xba*I (NEB), purified, and ligated. The resulting plasmid was amplified in *E. coli* and then inserted in MO10-WT by electroporation. The cmR construction was then introduced in MO10 containing pKDsgRNA-p15-kanR and grown at 30°C with 0.2% arabinose and 50 μg/ml of kanamycin, by electroporation at 1.25 kV, 5 ms, in 1 mm electroporation cuvettes. Mutants were selected on LB agar plates supplemented with 2 μg/ml of chloramphenicol at 37°C and verified by sequencing.

## Results

### Experimental evolution procedure

**FIGURE 1:**
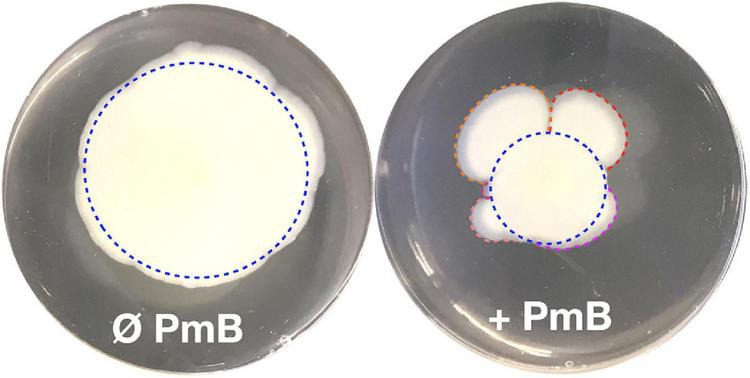
A flower-like pattern often appears in *V. cholerae* motility tests in the presence of polymyxin B. Motility assay on soft agar in the absence (Ø) or in the presence (+) of PmB. In the presence of PmB, the motility pattern of *V. cholerae* is not symmetrical as we would expect (blue dotted circles) but shows protrusions (red, orange, violet, salmon, and pink curved lines). In absence of PmB, the motility pattern is much less heterogenous than in the presence of PmB.

**FIGURE 2:**
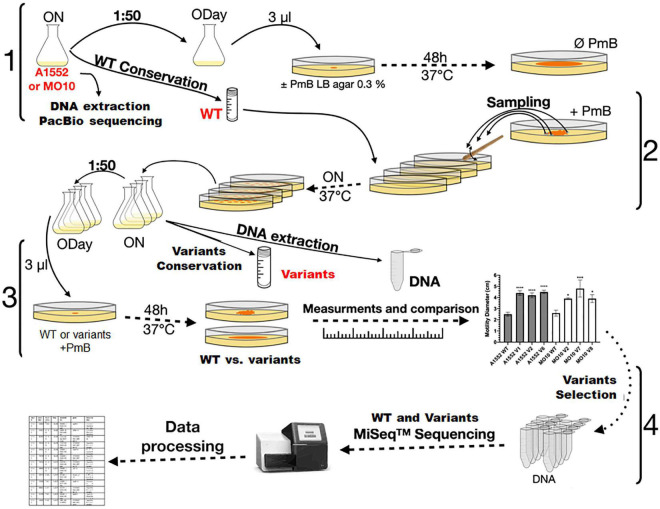
Experimental evolution design. *V. cholerae* A1552 and MO10 strains were grown overnight in LB broth. Genomic DNA from both strains was extracted and sequenced by Pac Bio. After dilution, the bacteria were grown to an optical density at 600 nm (OD_600 nm_) of 0.3 (ODay). Aliquots were spotted in technical triplicates on soft LB-agar supplemented or not with PmB. After incubation, samples of the different strains were taken from the edge of different protuberances of the flower-like patterns and streaked on LB agar. A colony from each of the different WT strains and their putative variants were grown overnight in LB broth and conserved at –80°C. The motility of the WT strains and variants was compared on soft agar plates supplemented with PmB. Motility diameters of the variants were measured and compared to those of their respective WT strains. The genomic DNA of selected hypermotile variants was extracted and sequenced by MiSeq.

**FIGURE 3:**
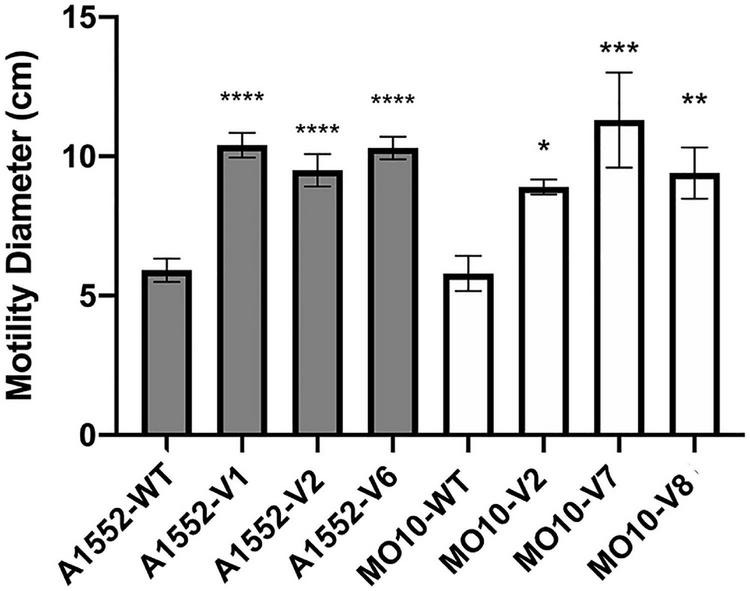
Variants of A1552 and MO10 present increased motility in the presence of PmB. Motility assay on soft agar supplemented with PmB after 48 h. Data are represented as mean ± SD of at least 3 independent experiments. Statistical significance between the variants’ motility diameter and their respective WT stains was tested by Dunnett’s one-way ANOVA. (*p < 0.05; **p < 0.01; ***p < 0.001; ****p < 0.0001).

In our previous study (Giacomucci et al., 2019), we observed a heterogenous pattern on soft agar with large protuberances in the presence of PmB (Figure 1). No protuberances were observed in the control without PmB. We hypothesized that they correspond to a mutation in the bacterial chromosome that compensates for the loss of motility in the presence of PmB. Therefore, we performed an experimental evolution to select for adaptative variants that gained motility in the presence of PmB (Figure 2). To do so, we plated the wild-type strains on soft agar supplemented with a subinhibitory concentration of PmB that causes no pore formation in the envelope of both strains used in this study (Giacomucci et al., 2019). After 48 h, we sampled bacteria at the edge of the protuberances and streaked them on LB medium to obtain isolated colonies. Two wild-type mother strains were used: *V. cholerae* O1 El Tor A1552 (A1552-WT) and O139 MO10 (MO10-WT). Eight samplings have been performed for each strain tested, and one colony representative of each sample has been selected and conserved for a total of 16 variants: A1552-V1 to V8 and MO10-V1 to V8. Then, we confirmed the motility phenotype of the variants in the presence of PmB (25 μg/ml) on soft agar. After 48 h of incubation, three variants of each strain were selected based on their increased motility in the presence of PmB (Figure 3). The variants selected are A1552-V1, A1552-V2, and A1552-V6 and MO10-V2, MO10-V7, and MO10-V8 from the A1552 and MO10 parental strains, respectively. Most of the remaining variants were less motile than the ones that were selected (*n* = 9), and one did not show any increase in motility at the confirmation step. As expected, the WT strains in the presence of PmB demonstrated reduced motility with a diameter of ∼2.5 cm. Without PmB, the motility pattern of the variants was comparable with the control, displaying a more circular pattern (data not shown) and a motility diameter of ∼4.3 cm on average (Giacomucci et al., 2019; Figure 3). Altogether, these results suggest that the variants have compensated for the loss of motility observed in the presence of PmB.

**FIGURE 4:**
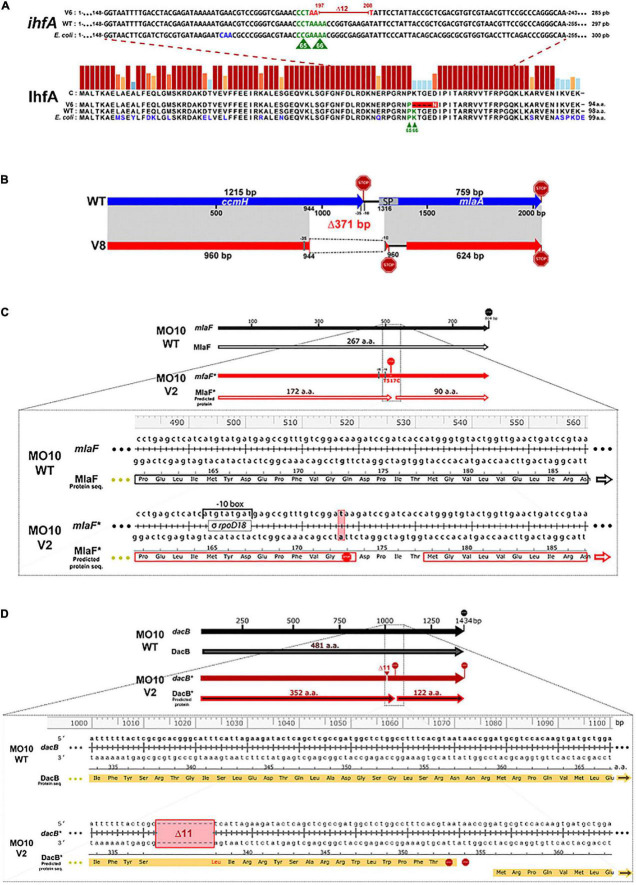
Identification of the mutations in the variant strains and their repercussions on the final product. **(A)** The deletion of 12 nucleotides in *V. cholerae* A1552-V6 suggests the loss of essential amino acid for IhfA function. Gene sequence (*ihfA*) and protein sequence (IhfA) alignments of *V. cholerae* A1552-V6 (V6), A1552-WT (WT), and *E. coli* MG1655 (*E. coli*). The IhfA protein sequences of *V. cholerae* A1552-WT and *E. coli* MG1655 share 94% similarity and 81% identity. P65 and K66 amino acids (in green), essential for IhfA function in *E. coli*, are also present in *V. cholerae* A1552-WT strain. However, in A1552-V6, the deletion of 12 nucleotides (197–208, in red) in the *ihfA* sequence leads to the substitution of a lysin residue in position 66 by an asparagine residue (in white) and the loss of 4 amino acids. **(B)** The deletion of 371 bp between *ccmH-1* and *vacJ* locus in MO10-V8 probably causes a loss of function of CcmH and VacJ. Schematic representation of *ccmH-1* (VchoM_01282) and *vacJ* (*mlaA*, VchoM_01281) locus alignment (in gray) between *V. cholerae* MO10-WT (blue) and MO10-V8 (red). The 371-bp deletion in MO10-V8 leads to the loss of ∼20% of *ccmH* 3’ end and a frameshift leading to the emergence of a new stop codon in the *vacJ’s* 5’-ORF region. The *vacJ* gene also lost important features, including its -35 and -10 promoter regions and the first 30 coding nucleotides, including the signal peptide (SP) sequence (nucleotides 1–51, codons 1–17). The *vacJ* predicted ORF in MO10-V8 starts 123 nucleotides (41 codons) forward and new putative –35 and –10 promoter regions for *vacJ* have been detected (see Supplementary Figure S1)**. (C)** A nonsense mutation in *mlaF* results in the loss of 26% of its original product. Alignments of *mlaF* gene sequence and MlaF protein translation sequence from MO10-WT and MO10-V2 (*). The substitution of the thymine-517 to cytosine in MO10-V2 resulted in the insertion of a premature stop codon (codon 173). MO10-V2 MlaF* has lost 95 amino acids (a. a.) corresponding to the ∼36% C-terminal end. A new ORF of 90 codons is created in the same reading frame, and a new promoter is predicted (refer to Supplementary Figure S2). **(D)** Deletion of 11 nucleotides in *dacB* in MO10-V2. Alignments of *dacB* gene sequence and DacB predicted amino acid sequence from MO10-WT and MO10-V2 (*). MO10-V2 had lost 11 nucleotides (1,013–1,023) resulting in a frame shift, causing the modification of 16 amino acids, the emergence of two early stop codons (STOP signs), and a new ORF starting at codon 360 of the WT sequence (refer to Supplementary Figure S3).

### Identification of the mutations

To determine the genes involved in the motility restoration of the variants in the presence of PmB, we performed comparative genomic analysis. To do so, we first sequenced the genome of the WT strains using PacBio technology. The variants were sequenced using a MiSeq technology and compared to the genome of their respective WT strain. The results of the comparative genomic analysis are presented in Supplementary Table S1 for A1552-V1, A1552-V2, A1552-V6, MO10-V2, and MO10-V8. The sequencing of MO10-V7 failed, and thus, we decided to pursue our investigation of the 5 variants with successful sequencing. Since the sequencing analysis was performed on DNA extracted from isolated variants, the only mutations that were considered are those with frequencies of 100%, which means that all the reads mapping this region identified the indicated mutations. The mutations were then confirmed by Sanger sequencing (Supplementary Materials and Methods).

The only mutation detected in the A1552 variants occurs in A1552-V6 and consists of a deletion of 12 nucleotides in *ihfA* (VC1222) (Figure 4A). This mutation results in the loss of 4 amino acids (67-70) and a mutation of one amino acid in position 66 (K66N). A comparison of the *ihfA* sequence in A1552 with its homolog in *E. coli* demonstrates that the sequence is highly conserved (94% similarity and 81% identity). Interestingly, the amino acids responsible for the IHF function in *E. coli* have been identified (P65 and K66) and are conserved in the wild-type sequence of A1552. Conversely, K66 is deleted in A1552-V6 (Figure 4A). Therefore, it is possible that the protein remains transcribed but with an attenuated functionality.

In MO10-V8, we noticed a deletion of 371 nucleotides in a region covering *ccmH* (VC2050) and *vacJ* (*mlaA*, VC2048) genes (Figure 4B). Regarding *vacJ*, which encodes a lipoprotein of the Mla (maintenance of lipid asymmetry) pathway, the first 17 codons were deleted, which results in the loss of the signal peptide for the secretion through the outer membrane as determined using SignalP 5.0 software (Almagro Armenteros et al., 2019). Therefore, it is expected that the mutation in *vacJ* induces a loss of function. Regarding *ccmH*, which encodes a c-type cytochrome maturation protein (Thony-Meyer, 1997; Braun and Thony-Meyer, 2005), the mutation results in the modification of four amino acids and the deletion of the 85 subsequent amino- acids in the C-terminal, which represents more than 20% of the sequence and the periplasmic domain of the protein.

In MO10-V2, we observed a point mutation resulting in the insertion of a stop codon in the *mlaF* (VC2520) sequence, which encodes an ATPase of the Mla pathway (Figure 4C). In addition, we observed in this variant an 11-nucleotide deletion in *dacB* (VC0632) in positions 1,013–1,023 (Figure 4D). This deletion results in a shift in the open reading frame and the emergence of a stop codon. In addition, DacB protein has lost ∼30% of its C-terminal residues. Therefore, it is very likely that *dacB* mutation in MO10-V2 results in a loss of function.

**FIGURE 5:**
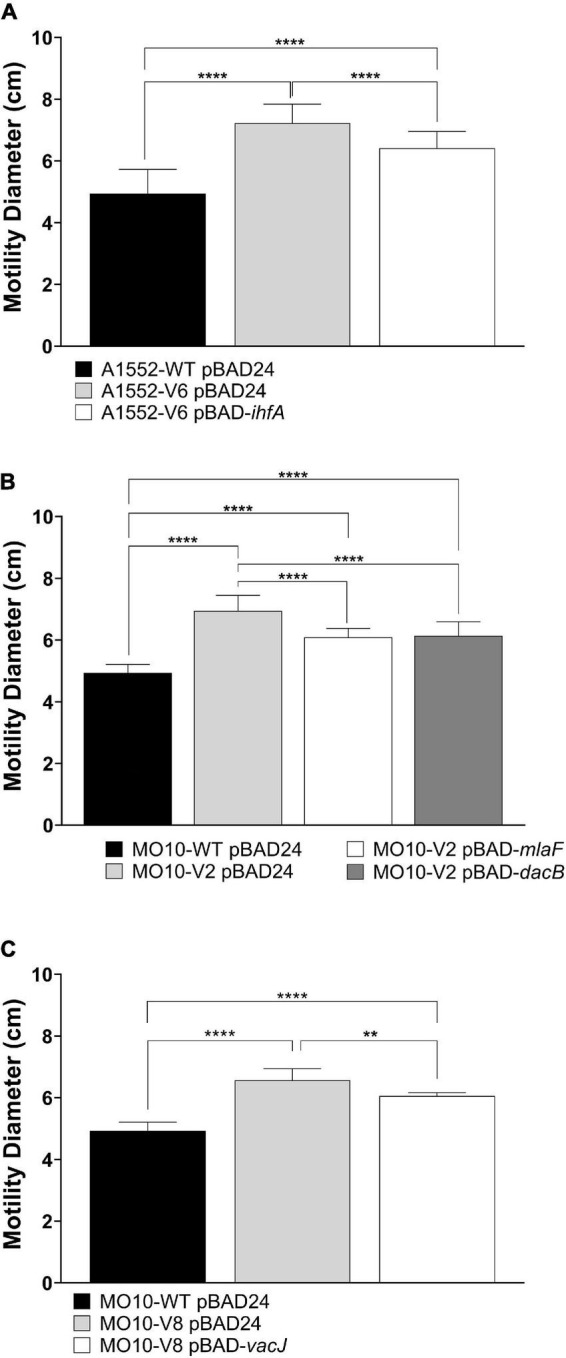
Effect of *ihfA*, *vacJ*, *mlaF*, or *dacB* overexpression on WT and variant strains’ motility on soft agar in the presence of polymyxin B. **(A)** A1552-WT and A1552-V6 carrying pBAD24 or pBAD24-*ihfA*, **(B)** MO10-WT and MO10-V2 carrying pBAD24, pBAD24-*mlaF* or pBAD24-*dacB*, and **(C)** MO10-WT and MO10-V8 carrying pBAD24 or pBAD24-*vacJ.* Data are represented as mean ± SD of at least three independent experiments (**p < 0.01; ****p < 0.0001).

To confirm that the mutations identified through the experimental evolution are responsible for the gain of motility, we complemented the variants with a WT copy of the genes of interest using the pBAD24 vector. The results demonstrate that the growth default of A1552-V8 is restored upon complementation (Supplementary Figure S4). Besides, the motility is partially restored after complementation for all the strains tested (Figure 5).

### Electron microscopy observations of the variants

**FIGURE 6:**
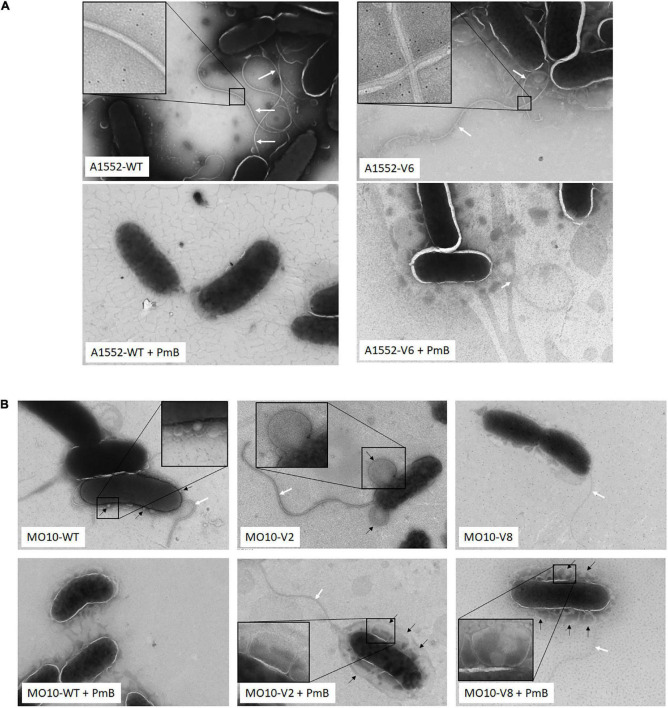
Electron microscopy images. **(A)** A1552-WT and A1552-V6 and **(B)** MO10-WT, MO10-V2, and MO10-V6. Bacteria were grown to an exponential phase in the absence and the presence of PmB (+ PmB). White arrows indicate the bacterial flagellum and black arrows indicate membrane vesicles.

To determine the impact of the mutations on the bacterial morphology, we observed the variants by electron microscopy. No major structural differences were observed between A1552-WT and A1552-V6 cells (Figure 6A). By looking closer into the flagellum’s structure, we observed that they both have an enveloped flagellum. However, while the A1552-WT filament is centered in the outer membrane envelope, the positioning of the A1552-V6 filament appears disorganized (Figure 6A). Regarding MO10-V2, we observed no difference in the flagellum and the cell structure while the flagellum of MO10-V8 appears thinner (Figure 6B). We also observed that MO10-V2 produces very large vesicles in comparison with MO10-WT (Figure 6B). In the presence of PmB, both MO10-V2 and MO10-V8 are entirely coated with large MVs, which is not the case with the MO10-WT (Figure 6B). In all variants, we can observe that the flagellum remains attached in the presence of PmB (Figures 6A,B) and not for the A1552-WT and MO10-WT strains, which is in line with the motility results.

### Determination of the role of the membrane vesicles in PmB titration and resistance

**FIGURE 7:**
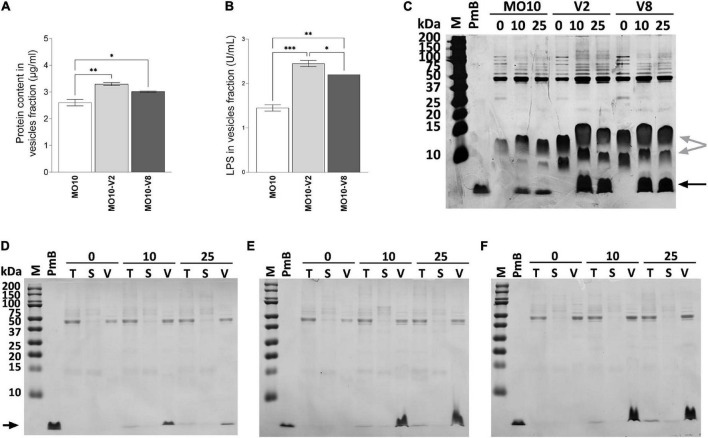
MO10-V2 and MO10-V8 variants produce more membrane vesicles and titer more polymyxin B than MO10-WT. The membrane vesicles were isolated by ultracentrifugation. The protein content **(A)** and LPS **(B)** of the vesicles isolated from MO10-WT (MO10) and its hypermotile variants, MO10-V2 and MO10-V8 cultures were quantified. Data represent mean values ± standard error of the mean from at least three independent experiments. (*p < 0.05; **p < 0.005; ***p < 0.0001). The polymyxin B (PmB) titration capacity of the vesicles from MO10-WT **(D)**, -V2 **(E),** and -V8 **(F)** was assessed by incubating the cell-free culture supernatant with 0, 10, or 25 μg/ml of PmB (0, 10, and 25, respectively). Samples from total culture supernatant (T), vesicles (V), and vesicles-free supernatant (S) were analyzed. Proteins were visualized on SDS-PAGE after Coomassie blue staining. PmB alone was used as control of the PmB size (black arrow). **(C)** The vesicle fraction from MO10-WT, -V2, and -V8 incubated with or without 10 or 25 μg/ml of PmB migrated on an SDS-PAGE gel. A silver nitrate coloration of the gel shows LPS (gray arrows) and PmB (black arrow). Gel pictures are representative of at least three independent experiments.

Because we observed the presence of large vesicles in MO10-V2 and MO10-V8 variants, we analyzed the vesiculation of these strains. To do so, the MVs of MO10-V2, MO10-V8, and MO10-WT were isolated by ultracentrifugation of the cell-free supernatant. The amount of MVs produced was estimated by quantification of the LPS and the protein content. MO10-V2 and MO10-V8 vesicle fractions are richer in LPS than the vesicle fraction of the MO10-WT, with MO10-V2 containing the largest amount (Figure 7B), suggesting an increase in the quantity of vesicles released. Moreover, a nitrate coloration of an SDS gel (Figure 7C) shows larger bands corresponding to the LPS size (gray arrows) in the vesicles of MO10-V2 and –V8 than in the MVs of MO10-WT. Similarly, the protein concentration of the vesicle fraction, estimated by the Bradford assay, showed an increase in MO10-V2 and -V8 in comparison with MO10-WT MVs (Figure 7A). MO10-V2 and -V8 complementation restored the protein concentrations in the vesicles to the MO10-WT level (data not shown). Similar results were obtained using a MO10Δ*vacJ*:cmR mutant in terms of MVs formation and PmB sequestration. The protein concentration increased by 1.68-folds, and we observed larger LPS bands on a sodium dodecyl sulfate–polyacrylamide gel electrophoresis (SDS-PAGE) gel upon *vacJ* mutation (Supplementary Figures S5A,B). Besides, the PmB sequestration assay showed that the MVs from MO10Δ*vacJ*:cmR sequestered more PmB than the MVs from MO10 (Supplementary Figure S5B).

**FIGURE 8:**
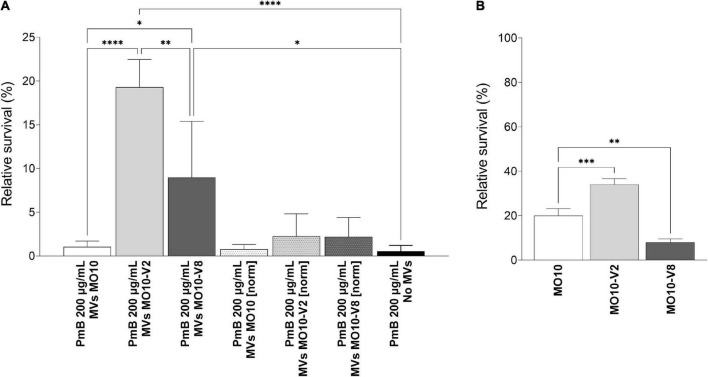
Relative survival to lethal concentration of polymyxin B. **(A)** Relative survival of MO10-WT in the presence of 200 μg/mL of PmB for 1 h, with or without MVs from MO10-WT, -V2, or -V8, normalized (MVs MO10-WT [norm], MVs MO10-V2 [norm], and MVs MO10-V8 [norm]) to the same concentration or not (MVs MO10-WT, MVs MO10-V2, and MVs MO10-V8). **(B)** Relative survival of MO10-WT, -V2, or -V8 after 30 min in the presence of a lethal concentration of PmB. Data are presented as mean ± standard error from at least 3 independent experiments. The % of survival was calculated in comparison with the condition without PmB (*p* ≥ 0.05; *p < 0.05; **p < 0.01; ***p < 0.001; ****p < 0.0001).

To further investigate the role of the MVs in the resistance of the MO10 variants to AMPs, we analyzed their ability to sequester PmB. To do so, we incubated the cell-free supernatant of MO10-V2, MO10-V8, and MO10-WT with 0, 10, and 25 μg/ml of PmB. After 1 h of interaction, we collected a sample of the total supernatant (T) and performed ultracentrifugation to separate the MVs (V) from the vesicle-free supernatant (S). The samples from the total supernatant (T), the vesicle fraction (V), and the vesicle-free supernatant (S) migrated on an SDS-PAGE electrophoresis gel. The PmB alone was used as control of the PmB size. The band corresponding to PmB size is not found in samples incubated without PmB (Figures 7D–F). We observed a band corresponding to the size of the PmB in the total supernatant (T) that is darker than in the vesicles-free supernatant (S). In addition, an intense band is observed in the vesicles fraction (V) (Figures 7D–F), suggesting that most of the PmB is associated with the MVs. In addition, we observed that the MVs isolated from an equal volume of MO10-V2 and MO10-V8 cell-free supernatants can sequester more PmB than the ones from MO10-WT, as shown by a larger band of PmB size (Figures 7D–F). To determine the ability of the MVs to protect the bacteria against PmB, we performed a protection assay using MVs. To do so, MO10-WT bacteria were incubated with the MVs from MO10-WT, –V2, and –V8, or without MVs, and a lethal concentration of PmB (200 μg/ml). We observed that the MVs from MO10-V2 and –V8 increased the survival of MO10-WT (Figure 8A), which is in line with the sequestration assay results. Conversely, the MVs from MO10-WT did not protect the bacteria from PmB. After concentration normalization of the MVs from MO10-WT, –V2, and –V8 according to the LPS dosage, the protection conferred by the MVs from MO10-V2 and –V8 was similar to that conferred by the vesicles of MO10-WT (Figure 8A). It indicates that the higher protection and sequestration of PmB by MO10-V2 and –V8 are probably due to the number of MVs produced rather than their composition. Altogether, our results suggest that the increased production of MVs by the MO10 variants allows more efficient sequestration of the PmB by the MVs, resulting in bacterial protection toward PmB.

We further investigated the resistance of MO10-WT and its variants to PmB by first determining the minimum inhibitory concentration (MIC) and observed no difference between the MO10-WT, –V2, and –V8 strains (data not shown). In parallel, we tested their ability to survive a lethal concentration of PmB. Our results show that MO10-V2 survival rate after 1 h is higher than the survival rate of MO10-WT (Figure 8B), indicating that MO10-V2 is more resistant to PmB than its parental strain. Conversely, the MO10-V8 appeared to be more sensitive to the PmB than MO10-WT (Figure 8B).

### Analysis of the flagellins genes’ expression

A1552-V6 presents a mutation on the predicted binding site of IhfA. IHF is an important regulator of virulence, colonization, and motility in *V. cholerae*. Thus, we wondered whether this mutation has an impact on the expression of the flagellar genes. To do so, we determined the relative expression of *flaA* and *flaE*, the first genes of both flagellin operons, in A1552-V6. We observed no difference in *flaA* and *flaE* expression in A1552-V6 in comparison with the parental strain A1552-WT (Supplementary Figure S6A), suggesting that the impact of the mutation in *ihfA* on the increased motility in A1552-V6 is not due to a modulation in the flagella-related genes. We also determined the expression of *flaA* and *flaE* in MO10-WT and its MO10-V2 and –V8 variants (Supplementary Figure S6B). Our results demonstrate a significant reduction of the flagellins’ expression in both variants.

## Discussion

To identify the genes that are important for motility in the presence of a sub-inhibitory concentration of PmB, we performed a short experimental evolution protocol. Experimental evolution has been widely used to validate evolutionary theories and to identify genes involved in responses to various stresses in bacteria (Mcdonald, 2019). In the latter case, *de novo* mutations driven by stresses led to the identification of genes that are important for survival in the presence of oxidative stress and antibiotics, or during a temperature shift (Hoffmann and Hercus, 2000; Justin et al., 2019). In this study, we used a targeted approach to identify the variants from single clones, which might have obscure other mutations. We identified mutations in genes potentially important for motility in the presence of PmB in two different strains of *V. cholerae*, i.e., A1552 (O1 El Tor) and MO10 (O139). These mutations in *vacJ*-*ccmH mlaF*, *dacB*, and *ihfA* have been identified in three variants, two in MO10, and one in A1552.

In our previous study, we demonstrated that the loss of motility in the presence of PmB was due to a high proportion of aflagellated bacteria (Giacomucci et al., 2019). Here, we show that there is no increase in the expression of the flagellins in the variants of both strains that could explain the gain of motility. However, if no difference is observed in A1552-V6, we observed a downregulation of both flagellin genes in the M10-V2 and –V8 variants in comparison with MO10-WT. This downregulation has no impact on their motility in the absence of PmB. *V. cholerae* has one single polar flagellum coated by an outer membrane sheath. In MO10-V8, the flagellum appears thicker in the electron microscopy pictures, whereas the flagellum of MO10-V2 and MO10-WT appeared to be alike. It has been demonstrated in *V. cholerae* that the structural integrity of the flagellum is linked to the composition of the outer membrane (Bari et al., 2012). The MO10 variants have a mutation in genes involved in the envelope biogenesis and maintenance. Thus, it is possible that the mutations affect the outer membrane composition and thereby the regulation of the flagellins’ expression.

We previously showed that the absence of the flagellum in the presence of PmB was not resulting in the repression of the flagellins genes, but likely to a default in the flagellum’s assembly or a flagellum miss-anchoring. In any case, the absence of the flagellum is related to envelope perturbations due to PmB, while no pores in the inner membrane were observed (Giacomucci et al., 2019). In this study, we identified 2 genes belonging to the maintenance of lipid asymmetry (Mla) pathway, i.e., *vacJ* (*mlaA*) and *mlaF*. VacJ is homologous to MlaA, an alpha-helical outer membrane lipoprotein, while MlaF is an ATPAse associated with the inner membrane, in complex with MlaD and MlaE. MlaC and MlaB are periplasmic and cytoplasmic proteins, respectively (Du Toit, 2019). It has been previously described in *E. coli* that the Mla pathway is essential for preserving the outer membrane lipid asymmetry by preventing phospholipids’ accumulation in the outer leaflet through an anterograde phospholipid transport (Malinverni and Silhavy, 2009; Du Toit, 2019) and is involved in the virulence toward silkworm (Nasu et al., 2022). The Mla pathway is important for antimicrobial tolerance in several gram-negative bacteria, including *Burkholderia* and *Pseudomonas* (Munguia et al., 2017; Bernier et al., 2018). In *V. cholerae*, it has been demonstrated that the Mla pathway is involved in MVs biogenesis and is important for serum resistance (Roier et al., 2016). The authors proposed that the hypervesiculation mediated by defects in the Mla pathway is a conserved bacterial mechanism to prevent phospholipids accumulation in the outer membrane (Roier et al., 2016). In this study, we demonstrate that the Mla pathway involved in hypervesiculation leads to the production of very large vesicles that can coat the bacteria in the presence of subinhibitory concentrations of PmB.

The deletion identified in MO10-V8 also affects the sequence of the gene adjacent to *vacJ*, i.e., *ccmH*, which encodes for a protein involved in a c-type cytochrome maturation. The cytochromes are important in bacterial metabolism as they function as electron transfer proteins. C-type cytochromes are characterized by the covalent liaison of the polypeptide with a heme (Kranz et al., 2009). In MO10-V8, a deletion of 371 nucleotides is observed, which represents ∼20% of the protein. Therefore, it is likely that CcmH has lost its function in this variant. In *V. cholerae*, there are 14 proteins containing a CXXCH motif and a signal peptide in N-terminal for the association with the membrane (Braun and Thony-Meyer, 2005). Among them, 7 have been characterized for their function in respiration in aerobic and anaerobic conditions, and one is a peroxidase probably involved in oxidative stress response (Braun and Thony-Meyer, 2005). CcmH is a part of the 6 other c-type cytochrome maturation proteins that are clustered together (*ccmA-I*) on *V. cholerae*’s chromosomes and are not required for growth under aerobic conditions in rich media (Braun and Thony-Meyer, 2005). To our knowledge, there is no evidence that *ccmH* is important for motility or membrane integrity, and its role in bacterial metabolism is probably very limited in our experimental conditions. Therefore, the implication of the deletion that covers both *ccmH* and *vacJ* sequences in bacterial motility recovery in the presence of PmB most probably relies on the inactivation of *vacJ*.

In another variant of the O139 strain MO10, a mutation has been observed in *dacB*, which encodes PBP4, a low molecular weight cell wall-synthesizing enzymes/penicillin-binding proteins (PBPs) involved in the peptidoglycan biosynthesis pathway (Typas et al., 2011). In *Vibrio parahaemolyticus*, *dacB* has a role in the formation of abnormal cell shape during the transition to a viable, but not culturable, state (Hung et al., 2013). In *E. coli*, low molecular weight PBPs also have a role in cell shape maintenance (Nelson and Young, 2001). The *dacB* mutant cells are wider and display localized transparent bulges at the poles (Yang et al., 2018). The authors also demonstrated that an accumulation of soluble peptidoglycan occurs in a *dacB* knockout mutant. Additionally, it has been demonstrated that peptidoglycan remodeling, especially the alteration in the peptidoglycan-outer membrane cross-linking, regulates the formation of MVs (Schwechheimer et al., 2015). This alteration involves the endopeptidase activities of PBP4 and another protein, Spr, under the regulation by NlpI. Altogether, the roles of *dacB* in cell shape maintenance, peptidoglycan biosynthesis, and vesicles formation pinpoint its importance in the cell wall remodeling that might be important for the stability of the flagellum in *V. cholerae*.

The role of vesiculation during *V. cholerae* infection in mammals has been investigated, and the results demonstrated that the Mla pathway is silenced at the early stages of infection to facilitate the outer membrane remodeling through hypervesiculation (Zingl et al., 2020). This remodeling confers resistance to AMPs and bile salts (Zingl et al., 2020). We previously demonstrated that the MVs of *Vibrio* can sequester the AMPs interacting with the outer membrane and the LPS, resulting in a reduction in the residual antimicrobial quantity interacting with the bacterial envelope (Duperthuy et al., 2013; Vanhove et al., 2015). In this study, we show that the MVs of both MO10 variants, MO10-V2 and MO10-V8, can sequester more PmB than the MVs of the parental strain. We further demonstrated that the MVs from MO10-V2 and –V8 can better protect the bacteria than their WT counterparts. This higher protection is most likely linked to the higher quantity of MVs produced, as protection assays using a similar quantity of MVs showed no differences between the MVs from the variants and the WT stain. Thus, the protection by the variants’ MVs is due to the hypervesiculation, as determined by LPS and protein quantification, and leads to the sequestration of a larger quantity of PmB. Besides, electron microscopy observations revealed that the MVs secreted by the MO10 variants are larger in size than those of the parental strain. Therefore, it is likely that the increased motility observed in the MO10 variants results from a reduction of the impact of the PmB on the bacterial cell membrane through MV sequestration, leading to increased stability of the flagellum.

While determining the ability of MO10-V2 and –V8 to resist PmB, we observed that more MO10-V2 survived to a lethal concentration of PmB, even though its MIC is similar to that of the WT strain. While performing MIC assays, bacteria are harvested at the early exponential growth phase and are heavily diluted (OD_600 nm_ = 0.0001). Thus, few MVs have been produced at this stage of growth and they have been diluted. Conversely, the survival assay was performed on bacteria at an OD_600 nm_ of 1 (midlog phase), without washing or diluting. Consequently, the MVs produced by the bacteria remained in the media during the assay. Thus, the increased survival of MO10-V2 to a lethal concentration of PmB is probably due to the sequestration of PmB by its MVs, which are more abundant than in MO10-WT cultures. Conversely, less MO10-V8 than MO10-WT appeared to survive while facing a lethal concentration of PmB, while its MIC is also similar to that of the MO10-WT strain. It has been previously reported that the MIC of PmB is not affected upon *vacJ* mutation in *V. cholerae* (Roier et al., 2016). It is, however, possible that the envelope integrity is slightly disturbed, not enough to observe a difference in MIC, but sufficient to observe a deficient survival to lethal concentrations of PmB. This result also implies that the quantity of MVs produced by MO10-V8 at the midlog phase is not sufficient to protect the bacteria from PmB, even though our protection results using MO10-V8 MVs from an overnight culture can protect MO10-WT. A thorough analysis of the dynamic of MV production in both MO10-V2 and –V8 would certainly increase our understanding of the differences between MO10-V8, –V2, and –WT in terms of survival to lethal concentrations of PmB and MV production.

**FIGURE 9:**
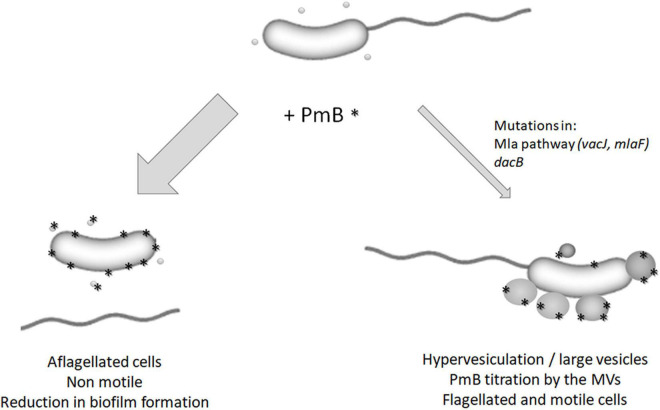
Model for the impact of polymyxin B on *V. cholerae* MO10. In the presence of subinhibitory and non-pore-forming concentrations of PmB, most of the bacterial cells are aflagellated, which results in non-motile cells and a reduction in biofilm formation (Giacomucci et al., 2019). However, some cells can adapt to the presence of PmB by mutating genes involved in the regulation of the vesiculation process (*mlaF*, *vacJ*, and *dacB*). It results in hypervesiculation and the formation of large MVs that can titer the PmB, thereby reducing its impact on the bacterial membrane and flagellum’s assembly and anchoring. These cells are flagellated and motile.

Altogether, the results of our previous study (Giacomucci et al., 2019) and the current study show that, in the presence of PmB, most of the *V. cholerae* cells are aflagellated and consequently non-motile (Figure 9). However, some cells can acquire mutations in important vesiculation regulatory pathways, leading to hypervesiculation through large MVs production and PmB titration. Consequently, the flagellum remains attached to the bacterial cell and motility is maintained (Figure 9). Thus, membrane remodeling and hypervesiculation are involved in *V. cholerae* adaptation to subinhibitory concentrations of PmB. However, since our study is based on a targeted approach by single variant isolation, it is difficult to determine to what extent the proposed mechanism is common at the population level. A global approach using parental and evolved populations would answer this question.

The only mutation identified in the O1 El Tor strain A1552 is a deletion of 12 nucleotides in *ihfA*. *ihfA* encodes IhfA, one of the IHF subunits, together with IhfB. IHF is a histone-like DNA-binding protein that regulates many functions in bacterial cells, including transcription, replication, and virulence (Freundlich et al., 1992). In *V. cholerae*, IHF is essential for conjugation and virulence genes’ expression (Mcleod et al., 2006). The deletion of 12 nucleotides likely results in the attenuation of IHF function since one of the two essential amino acids for DNA binding has been deleted. It has been demonstrated in a *V. cholerae* O1 El tor that IHF binds to *rpoN* and *flrA*, two major regulators of flagellar genes’ expression (Wang et al., 2012). The binding of IHF to *rpoN* and *flrA* promoter regions leads to a limitation of H-NS binding to these regions (Wang et al., 2012). The role of H-NS as a negative regulator of *rpoN* and *flrA* has previously been described (Prouty et al., 2001). Thus, an IHF inactivation would lead to the binding of H-NS and eventually to the repression of the flagellum-related genes. However, we noticed that the IHF-binding site in the promoter region of *rpoN* is absent in A1552. We previously demonstrated that the expression of the flagellin subunits is not reduced in the presence of PmB (Giacomucci et al., 2019), and in this study, we observed no difference in flagellins’ expression in A1552-V6. Since IHF-binding sites have been identified all over the bacterial genome (Prieto et al., 2012; Reverchon et al., 2021), it is reasonable to hypothesize that the mechanisms behind the gain in motility in the presence of PmB involving *ihfA* mutation are not related to the flagellum itself. As stated above, the loss of motility in the presence of PmB is likely due to a structural defect in the flagellum or the cell envelope. It has been recently demonstrated that a deletion of *ihfA* in *Dickeya*, a bacterium belonging to the *Enterobacteriaceae*, results in strong transcriptomic modifications, reflecting a “survival mode” associated with cell envelope modifications (peptidoglycan, porins, lipopolysaccharide, and exopolysaccharides) (Reverchon et al., 2021). In addition, the expression of major porins of *E. coli* is controlled by IHF (Huang et al., 1990; Ramani et al., 1992). In *Salmonella enterica*, an *ihfA* mutation induces the expression of genes involved in peptidoglycan and lipopolysaccharides biosynthesis (Mangan et al., 2006). Therefore, it is possible that a mutation in *ihfA* influences the expression of genes that strengthen the bacterial envelope, leading to the retention of the flagellum in the presence of PmB.

## Data availability statement

The datasets presented in this study can be found in online repositories. The names of the repository/repositories and accession number(s) can be found in the article/Supplementary material.

## Author contributions

SG, AM-D, and MD: conceptualization. SG, AM-D, and AV: data curation and formal analysis. SG, AM-D, AV, and HJ: investigation. SG and AM-D: methodology. AV: software. SG, AM-D, and MD: validation and visualization. AM-D, SG, AV, and MD: writing – original draft. SG, AM-D, AV, HJ, and MD: writing, reviewing, and editing. MD: resources, supervision, funding acquisition, and project administration. All authors contributed to the article and approved the submitted version.
