# Supplementary material for: Experimental evolution of Vibrio cholerae identifies hypervesiculation as a way to increase motility in the presence of polymyxin B
Source: Front Microbiol. 2022 Aug 22;13:932165. doi: 10.3389/fmicb.2022.932165 (PMC9454949; doi:10.3389/fmicb.2022.932165)
Supplement: Supplementary file 1 [file Data_Sheet_1.docx]

Supplementary Material

# Supplementary Table

**Table S1 Mutations identified in A1552and MO10 variants**

| **Variant** | **Chromosome** | **Position** | **Mutation** | **Annotation** | **Gene name or locus^a^** | **Description** |
| --- | --- | --- | --- | --- | --- | --- |
| A1552-V1 | No mutation identified (frequency <100%) | | | | | |
| A1552-V2 | No mutation identified (frequency <100%) | | | | | |
| A1552-V6 | Chr 1 | 1029617 | Δ12 bp | coding (187198/297 nt) | *ihfA →* | Integration host factor subunit alpha |
| MO10-V2 | Chr 1 | 375972 | C→T | Q173* (CAA→TAA) | *mlaF →* | Intermembrane phospholipid transport system ATPbinding protein MlaF |
| MO10-V2 | Chr 1 | 2467074 | Δ11 bp | coding (10131023/1446 nt) | *dacB →* | DalanylDalanine carboxypeptidase DacB |
| MO10-V8 | Chr 1 | 875871 | Δ371 bp |  | *ccmH-mlaA* | Cytochrome c-type biogenesis protein CcmH - Intermembrane phospholipid transport system lipoprotein MlaA |

a: Gene or locus name corresponding to *Vibrio cholerae* A1552 chromosome 1 (CP072847.1) or *Vibrio cholerae* MO10 chromosome 1:CP072849.1

# Supplementary Figures


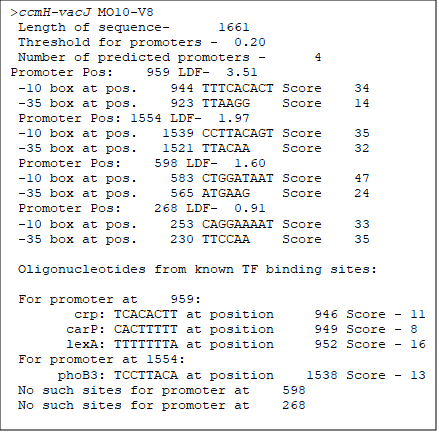


**Supplementary Figure 1**. **BPROM prediction for MO10-V8 *vacJ*promoters.** In MO10-V8, mutations in ccmH and vacJ resulted in the apparition of a new ORF at position +624 (see Supplemental material and methods).


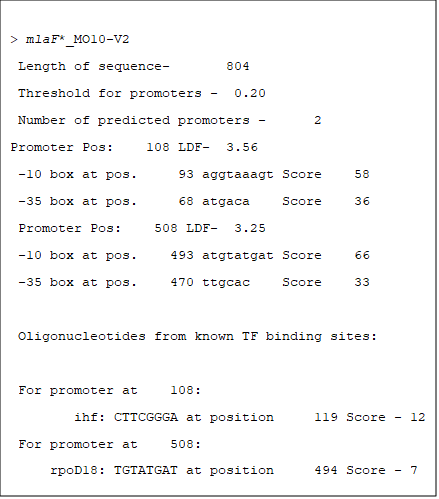


**Supplementary Figure 2. BPROM prediction for MO10-V2 *mlaF* promoters.** In MO10-V2, a mutation in *mlaF* resulted in the apparition of a new ORF at position +532 (see Supplemental material and methods).


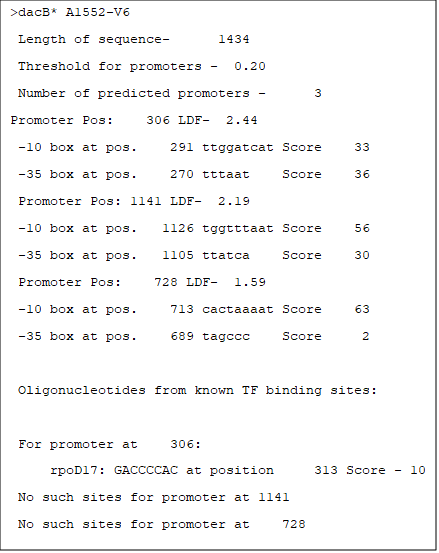


**Supplementary Figure 3. BPROM prediction for A1552-V6 *dacB* promoters.** In A1552-V6, a mutation in *dacB* resulted in the apparition of a new ORF at position +1067 (see Supplemental material and methods).


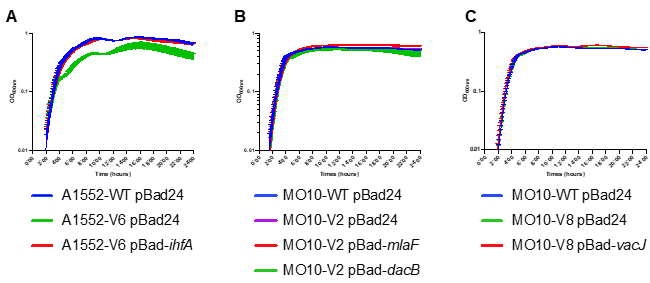


**Supplementary Figure 4. Growth curves in LB medium of A1552, MO10, their variants and complemented strains.** A) A1552-WT carrying pBAD24 (blue) and A1552-V6 mutant carrying pBAD24 (green) or pBAD24-*ihfA* (red), B) MO10-WT carrying pBAD24 (blue) and MO10-V2 carrying pBAD24 (purple), pBAD24-*mlaF* (red) or pBAD24-*dacB* (green), and C) MO10-WT carrying pBAD24 (blue) and MO10-V8 carrying pBAD24 (green) and pBAD-*vacJ* (red). Results are presented as OD_600nm_ ± SD and correspond to three technical replicates within three biological replicates.

Supplementary Figure 5. MO10Δ*vacJ*::cmR (MO10Δ*vacJ*) produces more vesicles that sequester more polymyxin B than MO10. A) The protein content of the vesicles isolated from MO10-WT and MO10Δ*vacJ*::cmR (MO10Δ*vacJ*) culture supernatant was quantified by a Bradford assay. Data represent mean values ± standard error of the mean from at least 3 independent experiments (*p<0.05). B) The polymyxin B (PmB) titration capacity of MO10-WT and MO10Δ*vacJ*::cmR (MO10Δ*vacJ*) was assessed by incubating the cell-free supernatant with 0 or 10 µg/mL of PmB. After incubation, the MV fraction was collected by ultracentrifugation and migrated on SDS-PAGE gel. A silver nitrate coloration of the gel shows LPS (gray arrow) and PmB (black arrow). Gel pictures are representative of at least 3 independent experiments.

 
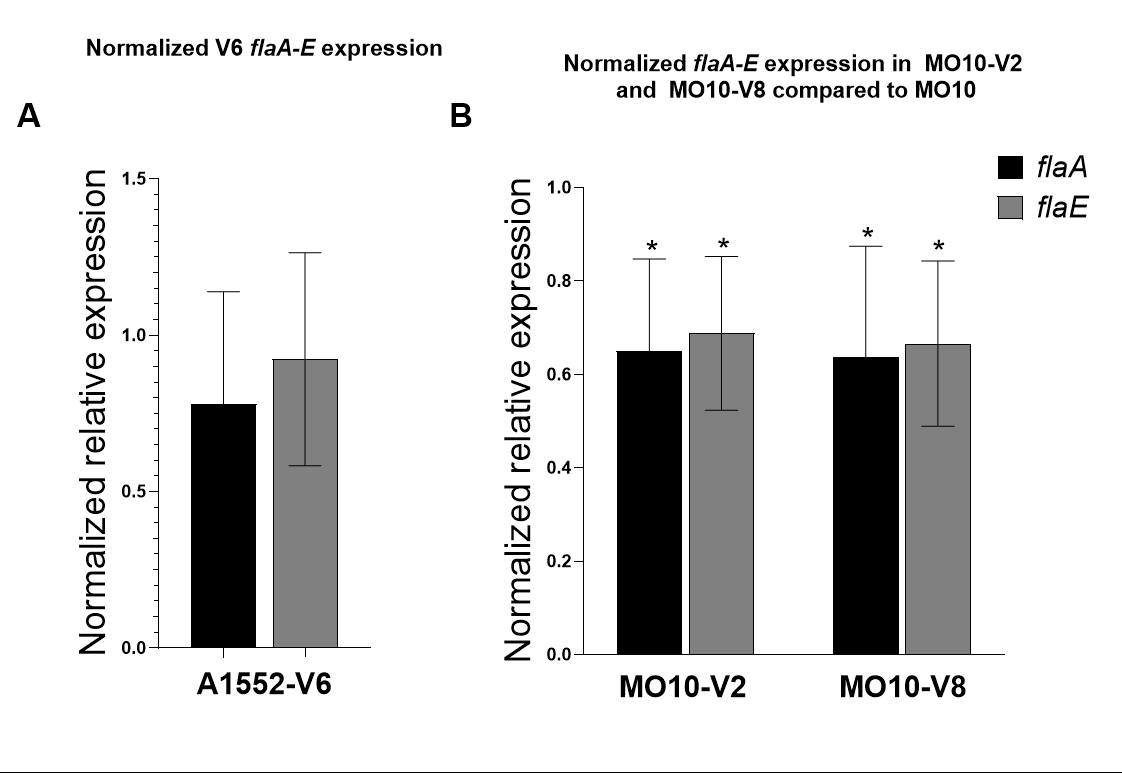


**Supplementary Figure 6. Normalized relative expression of *flaA* and *flaE.* (A)** A1552-V6 in comparison with the parental strain A1552-WT **B.** MO10-V2 and -V8 in comparison with the parental strain MO10-WT. Data represent mean values ± SD from at least 3 independent experiments.

# Supplemental Material and methods

A1552-V2, MO10-V2 and MO10-V8 mutations

Full open reading frame sequences of *ihfA*, *dacB*, *ccmH-vacJ*and *mlaF*verified by Sanger sequencing. Sequences start at first nucleotide of indicated gene ORF.

>*ihfA* in A1552-V6

ATGGCGCTCACAAAGGCCGAATTGGCTGAAGCCCTGTTCGAACAGCTCGGCATGAGCAAGCGGGATGCCAAGGATACGGTTGAGGTGTTTTTTGAAGAAATTCGTAAAGCACTCGAAAGTGGCGAACAGGTAAAACTCTCCGGTTTTGGTAATTTTGACCTACGAGATAAAAATGAACGTCCGGGTCGAAACCCTAATATTCCTATTACCGCTCGACGTGTCGTAACGTTCCGCCCAGGGCAAAAATTGAAAGCCCGTGTCGAGAACATCAAAGTCGAAAAATAA

>*dacB* in MO10-V2

ATGCTTTTTCGCTTCATACCTGTTTGGTTACTCTCTATTAGCGGTTTTCTAATAGCCTCTCCGATTTACGCACAAACACCATTGACTGCCGCAACGACTAAACTTCCTCAAGGGGCACGTTATAGCCTATTGATTGAAGATGTCGCATTACAGCAGAACACTCTCGAACTCAATACTCATCTGTACTATCCCCCCGCTAGCACCCAGAAGATTTTGACGGCACTCGCCGCAAAATTAGAACTGGGTGATAAGTTTCGCTTTCACACTGATTTAATGCGTTCAGGGCAAGATTGGATCATTCGCTTTTCAGGCGACCCCACCCTGACCACCGCAGATTTAACGACATTGCTCAAAGCGATGAAAGCGCAAAGTGGCGGTAGGATTGAGGGCGATTTGTGGCTGGATAATAGTGTATTTAGTGGATATGAGCGTGCGGTAGGCTGGCCATGGGATATTTTAGGCGTCTGCTATAGCGCCCCAGCCAGTGCCATCAACCTCGATGCTAACTGTATCCAGGCGTCTATTTATACCGAACCACAAGGTAAAACGCGCGTTTATGTACCAGAGCACTACCCTGTGCATGTTCAGTCGCAAGCCATCAGCGTGACACAGAGCGAACAAGAGAGTTTACTGTGTGACTTAGAACTGACGGCAACGCCTGAGAATCACTATACGCTGGATGGCTGTTTAGCCCTACGAGACAAACCCTTACCACTAAAATTTGCGGTGCAAGATACTGGGATCTACACCCAGCGAGTGGTCTATCGTCTCCTCAGCCAGCTAAACATCGAGCTCAAAGGGAAGATAAAAGTCGGTAAAGCAAATACCAAACAAGCGCAGAAAATCGCTTCTCATCACTCCCAGCCGCTGCCTGTGTTACTGAAAACCATGTTGCAAGAGTCCGACAACCTGATCGCCGATACCTTGACCAAAGCCTTGGGACACCGTTTTTACTCTCAACCCGGTAGCTTTACCAACGGAACACAAGCCATTAAACAGATTTTTTACTCGCTCATTAGAAGATACTCAGCTCGCCGATGGCTCTGGCCTTTCACGTAATAACCGGATGCGTCCACAAGTGATGCTGGAAACTCTTCGCTACCTTTATCAGCACGAAGCTGAGCTTGGTTTAATTGCTATGCTGCCTTCAGCGGGAGAATCGGGCACTTTGCAATATCGACGCAGTATGCGTGCGCCGCAAATCAGTGGCCAAATTAAAGCGAAAAGTGGTTCACTTTATGGCACTTACAATATGGCGGGCTTTGTGATGGACGAAAATCAGCGCCCTAAGACTCTGTTTGTTCAATTCGTCACCGACTATTTCCCTCCGAGATCCAATCCTGAGGTAGCGGTTGAGCCGCCGATTATCCAGTTTGAAACTCAGCTCTATCAAGAGCTTATTCAGTTTAATCGTTTGGCATCTAAGCCAAACTA

>*ccmH*-*vacJ* in MO10-V8

ATGTGGATGTTTTGGATCTCGACCCTATTACTGGTGGCGATTGCGGTGGTTTTCGTCATCATTCCGTTTATTCAAAAGCGTGCGAATAACGATCAGGCTTTGCGCGATGAGCTGAATAAAGCGTTTTACAAAGACCGCTTGAAAGAGCTTGAAGAGGAAACCGAAGAAGGCATTGTTGCCGATCAACAAGATTTGATTGCCGACTTAAAACAGACTCTGCTTGACGACATTCCAACCCAGCAAAAACATCAGCAGGAAAATCGTGTTTCACTGTGGATGGTTGCCCTGCCTTCAGTATTGTTGGTAGTCGGATTGAGTTATGCGCTGTACGCCAAGTTTGGTCACTATCAGCATGTTCAGGCTTGGCAGCAAGTGTCAGCACAACTGCCTGAATTGTCAAAAAAACTCATGTCGCCACAAGCGGAACTCAGTGACGAAGAGATGAATGATTTGACGTTGGCACTGCGCACTCGACTGCATTATCAGCCTGATGATGTTACCGGTTGGTTGTTGCTGGGTCGGATTGGCCTTGCTAATCGCGATCTGGAAACCGCGATTGGCGCGATGAAGAAAGCTTTTGCTCTGGATAATGAAGATCCGGATGTGAAATTTGGTTACGCACAAGCTTTGATGCTTTCGAATGATCCTGTCGACCAGCAAGAAGCGAAGTCGATTCTGCTCAAGTTAGCCCAACGTGGTTATGCTGATTTACGCGTCTATTCATTATTGGCGTTTGATGCTTTTGAAAGTGGAGATTTTCCTGCTGCAATCAAGTACTGGAGTTTGATGCAACAAGCGATTGGTCCTGACGATGCTCGTTATGAGATGCTCAGCCGCAGTATTGAAAGCGCTCGTAAGAGAATGGGCGAGGGCATGGCAGAGGGTCAATCGGTGAAAGTCACCATTAATCTAGGCGAGCAGGTTAAGGTTGATCCTAACGCAGTTTCACACTTTTTTTAGTTGGGTGCAGCAGTGCACCTGATGACTCCTCCCCTCATTCGCAGGTGAACGATCCTCTGGAAAGTTTCAACCGGCAAATGTGGACAATTAACTATGACTACCTAGACCCTTATGTGGTGCGTCCGGTCTCTCTATTTTATGTCGGTTATGTACCTAAGCCTGTACGCAGTGGCATTGCCAACTTCCTCTCTAACTTAGACGAGCCTGCCAGCATGGTGAATAACCTGCTGATGGGCAATGGGACAAAAGCGGTCGATCACTTTAATCGTTTTTGGATTAATACCAGCTTTGGTTTACTCGGTTTGATTGATATCGCTTCTGAAGCAGGCATCAAAAAATACGATGATAAGGCGTTCAGTGATGCGGTAGGCCATTACGGTGTGGGCAATGGCCCCTATTTAATGGTCCCAGGTTATGGTCCCTATACGGTACGCGAAGTGACCGATGTGGTGGATGGCATGTATTTCCCGCTTGCCTATCTCAATATCTGGGCTGGGGTCGGCAAATGGGCACTTGAAGGCATGGAAACGCGCGCTGCGTTAGTTTCGCAAGAGGCCTTATTACAAGACTCACCTGATCCTTACAGTTTGGCTCGCGATGCTTATCTCCAACGGCAAGCTTTCAAAGCGGAGATCCAAGTGGATGACTATGACCCTGAGGAAGAAGAGTATCTCGATGAGTATTTAAATGAAGGGTTATGA

>*mlaF* in MO10-V2

ATGTCTCAATCTGACTTAGTCACCATCAAAAATTTGCGTTTTTCGCGCTCGCAGCGCGTCATTTTTGATGACATAGATCTGCATGTCCCCAAAGGTAAAGTGACAGCAATTATGGGGCCTTCGGGAATCGGTAAAACCACACTGCTGCGTTTGATCGGCGGTCAACTCCTGCCAGAACAGGGAGAGATCTGGTTCGATGGTGAAAATATTCCCACCCTCAGTCGCCGCAAACTGTATCGTGCTCGTAAGAAGATGAGCATGCTGTTCCAATCAGGCGCGCTGTTTACCGATCTTAATGTGTTTGACAATGTGGCTTACCCATTGCGCGAGCATACCGAACTTGATGAAGCCATGATTAAAACCTTGGTGCTGCTGAAATTAGAGGCGGTTGGACTGCGTGGTGCTGCGTATTTAATGCCTAGTGAGCTTTCAGGCGGTATGGCGCGCCGCGCCGCACTGGCAAGGGCTATTGCACTCGATCCTGAGCTCATCATGTATGATGAGCCGTTTGTCGGATAAGATCCGATCACCATGGGTGTACTGGTTGAACTGATCCGTAACCTTAATCGAGCCTTGGGTGTCACCTCTGTGGTGGTATCGCACGATGTACCGGAAGTGATGAGCATTGCGGATTGGGTTTATCTGTTGGCCGATGGTAAGGTGATTGCGCAAGGTTCACCTCAAGCATTGCGCGACAACCCTGATCCGCGTGTACAACAATTTTTATGCGGCGATGCAGATGGCCCTGTGCCATTTCGTTTTCCTGCGCAGCCGATAGAACAGGAGCTGTTTAGTGCTAAATGA
